# Induction of *Escherichia coli* Into a VBNC State by Continuous-Flow UVC and Subsequent Changes in Metabolic Activity at the Single-Cell Level

**DOI:** 10.3389/fmicb.2018.02243

**Published:** 2018-09-25

**Authors:** Shenghua Zhang, Lizheng Guo, Kai Yang, Yin Zhang, Chengsong Ye, Sheng Chen, Xin Yu, Wei E. Huang, Li Cui

**Affiliations:** ^1^Key Lab of Urban Environment and Health, Institute of Urban Environment, Chinese Academy of Sciences, Xiamen, China; ^2^College of Geography, Fujian Normal University, Fuzhou, China; ^3^Department of Engineering Science, University of Oxford, Oxford, United Kingdom

**Keywords:** continuous-flow UVC, VBNC, induction, resuscitation, metabolic activity

## Abstract

A viable but non-culturable (VBNC) state of bacteria induced by disinfection in water treatment poses serious health risks because of possible resuscitation of VBNC cells during transportation. In this study, a setup using continuous-flow ultraviolet (UVC) irradiation ranging from 0 to 172.2 mJ cm^-2^ was designed to simulate real-world disinfection in both drinking water (SDW) and reclaimed water (SRW) treatment plants. A systematic investigation of UVC-induced VBNC bacteria, including occurrence, resuscitation, and time-dependent recovery of metabolic activity during post-incubation, was conducted. Different techniques including two new ones of “single cell culture” and D_2_O-labeled single-cell Raman spectroscopy were employed to gain comprehensive insights into VBNC cells. Heterotrophic plate counts (HPC) and 5-cyano-2,3-ditoyl tetrazolium chloride flow cytometry (CTC-FCM) assay demonstrated that exposure to continuous-flow UVC can induce *E. coli* into a VBNC state. Membranes integrity and 16S rRNA transcription level of VBNC bacteria were demonstrated to be unaffected by UVC exposure even at a high dose of 172.2 mJ cm^-2^. Resuscitation of VBNC bacteria was identified in a more accurate way based on “single cell culture.” Finally, time-dependent evolution of metabolic activity of UVC-treated cells during post-incubation was examined by D_2_O-labeled Raman spectroscopy at a high-resolution of single-cell level. C-D Raman bands resulting from incorporation of D_2_O-derived D into bacterial biomass were used as a sensitive and quantitative indicator of bacterial metabolic activity. A lower UVC dose, longer post-incubation time, and higher initial number of bacteria were demonstrated to result in a faster recovery of metabolic activity. Heterogeneous metabolic activity and subpopulation with higher metabolic activity were also revealed by single-cell Raman, even for UVC-treated cells losing cultivability. The comprehensive assessment of VBNC bacteria in UVC-disinfected drinking and reclaimed water points out treatment deficiencies of UVC and the necessity to develop more effective strategies to eliminate VBNC cells.

## Introduction

Viable but non-culturable (VBNC) state are bacteria that are living but unable to form colonies when cultured in bacteriological media, but capable of resuscitation under precise stimuli such as nutrients and temperature (Oliver, 2000; Moreno et al., 2007). Presence of VBNC bacteria in public health-related fields poses a great risk to human health, because their occurrence underestimates the number of total viable bacteria and thus associated health risk, in addition, their ability to resuscitate also endangers human life. Water treatment systems including drinking and reclaimed water treatment are closely related to human life. More and more work has indicated that routine disinfection techniques used in water treatment can potentially induce bacteria into a VBNC state, such as chlorine, ozone, and ultraviolet (UVC) light (Oliver et al., 2005; Zhang et al., 2015; Orta de Velásquez et al., 2017).

UVC disinfection is widely used for water treatment because it is more effective against cysts of *Cryptosporidium* and *Giardia* than chlorination and ozonation without producing disinfection byproducts (Belosevic et al., 2001). However, UVC-induced VBNC state of bacteria are not well understood and related studies are very scarce. Our previous study identified the possible public health risk of UVC radiation as a disinfection technology, and concluded that *Escherichia coli* and *Pseudomonas aeruginosa* can be induced into a VBNC state by 254 nm UVC irradiation. This preliminary work used simple static UV irradiation, but did not take continuous-flow UVC disinfection and time points for water discharge used in real-world water treatment into consideration. New experimental setup simulating real-world UVC disinfection is highly desired, in order to provide a more realistic estimation of VBNC cells induced by UVC. In addition, technique advances are also very important in promoting the understanding of VBNC cells and their risks, such as development of a more accurate way to analyze resuscitated cell and single-cell level analysis of heterogeneous physiology evolution of VBNC cells.

Many methods have been used to determine the viability of bacteria, which is an important feature of VBNC cells. Bacterial cells carry out numerous processes essential to their survival, including respiration, cell membrane integrity, ATP synthesis, enzymatic reactions involved in metabolic processes, DNA replication, RNA transcription, and protein translation. There is no uniform or single criterion to define bacterial variability, instead, bacterial cell can be regarded to be ‘viable’ if they maintain essential processes for their survival presented above (Manina and McKinney, 2014). Different techniques have been used to assess cellular viability from different aspects of cellular physiology and metabolism. For instance, respiratory activity can be characterized by respiration-dependent reduction of 5-cyano-2,3-ditoyl tetrazolium chloride (CTC) (Junge et al., 2004). CTC can be reduced via electron transfer chain to form a substance emitting red fluorescence. The intensity of red fluorescence reflects the amount of bacteria with active respiration. The further combination with flow cytometry assay (CTC-FCM) provides a way to detect viable cells at single-cell level (Li et al., 2013). CTC-FCM has been applied to study the regrowth of pathogenic bacteria in VBNC state induced by chlorination in reclaimed water with a long retention time (Li et al., 2013). Membrane integrity can be assessed with LIVE/DEAD *Bac*Light bacterial viability kit that contains two nucleic acid stains of membrane-permeable SYTO9 (green) and only damage membrane-permeable propidium iodide (red). In addition, propidium monoazide quantitative polymerase chain reaction (PMA-qPCR) provides another way to quantify viable bacteria by characterizing membrane integrity, based on the principle that binding of PMA to DNA of membrane-damaged cells can strongly inhibit DNA amplification (Zhang and Yu, 2010). PMA-qPCR has been used to enumerate VBNC *Legionella pneumophila* induced by heat (Slimani et al., 2012). Reverse transcription qPCR (RT-qPCR) combined with real-time PCR can qualitatively determine gene expression and precise number of related cells by quantifying RNA (Lleò et al., 2000). Combining RT-qPCR assay with heterotrophic plate counts (used for culturable cells) has been developed to quantify Enteric bacterial pathogens in the VBNC state in sewage sludge (Jiang et al., 2013).

Despite these progresses, it is still difficult to distinguish the contribution to treatment failure from the regrowth of residual culturable cells or the resuscitation of VBNC bacteria. Some studies serially diluted culturable bacteria to an extremely low level and regarded the re-growth as resuscitation of VBNC cells. However, culturable cells were still there, their contribution cannot be totally eliminated (Kaprelyants et al., 1994; Whitesides and Oliver, 1997). The other study applied antibiotics at the minimal inhibitory concentrations (MICs) to kill culturable bacteria; the remaining VBNC bacteria with greater resistance were employed to identify resuscitation (Lleò et al., 2007). However, the possibility that VBNC bacteria were also killed and meanwhile persistent cells showed up under antibiotic treatment could not be excluded, bringing in some error in distinguishing VBNC cells. In this study, a more accurate way based on comparison of the number of culturable cells in UV-treated samples with that of regrowth cells during post-incubation (including both residual culturable cells and VBNC bacteria) determined by single-cell culture was used to identify resuscitation.

Another important aspect of VBNC cells is their heterogeneous recovery of metabolic activity during resuscitation. An emerging novel technique of heavy water (D_2_O)-labeled single-cell Raman can potentially provide new insights into the recovery process during post-incubation. Single-cell Raman spectroscopy is a non-destructive method for molecular profiling based on vibrational frequencies of chemical bonds in individual cells. When combined with stable isotope labeling, incorporation of a stable isotope by viable cells can induce obvious Raman band shifts due to substitution of light atoms with heavy isotopes in chemical bonds, providing a good indicator for metabolic active cells (Huang et al., 2004; Li et al., 2012; Berry et al., 2015; Wang et al., 2016; Cui et al., 2017, 2018; Song et al., 2017). A hydrogen atom (H or D) is indispensable in the biosynthesis of diverse biomolecules in bacteria. Incorporation of a stable D isotope from D_2_O can cause a dramatic shift in the C-H bond from 2800–3100 cm^-1^ to a C-D bond at 2040–2300 cm^-1^. The resulting intensity ratio of (C-D)/(C-H + C-D) can act as a semi-quantitative indicator of metabolic activity of a microorganism. D_2_O-labeled Raman spectroscopy has been used to detect the general physiological activity of bacteria and their heterogeneous response to carbon sources (enhancement) or antibiotics (inhibition) at the single-cell level (Berry et al., 2015; Song et al., 2017; Tao et al., 2017). These advantages indicate great potential of single-cell D_2_O-labeled Raman in studying heterogeneous metabolic activity of VBNC bacteria during post-incubation.

In this study, in order to fully understand the occurrence and risk of VBNC bacteria, we constructed a continuous-flow 254 nm UVC equipment to simulate the real-world disinfection used in both drinking water (SDW) and reclaimed water (SRW) treatment systems. Physiology and metabolism of UVC-induced VBNC state of bacteria were characterized by different methods including HPC (culturability), CTC-FCM (respiratory activity), PMA-qPCR (membrane integrity), and RT-qPCR (gene expression). A new method of “single-cell culture” was applied to identify resuscitation in a highly accurate manner. Absolute numbers and ratios of VBNC bacteria were also calculated to evaluate the risk of water after UVC disinfection. D_2_O-labeled Raman spectroscopy was further employed to investigate time-dependent heterogeneous changes of metabolic activity of VBNC cells during post-incubation at single-cell level. The comprehensive investigation of VBNC bacteria from occurrence, resuscitation, and metabolic activity evolution during post-incubation will promote recognition of treatment deficiencies and development of more effective strategies to mitigate risk of VBNC bacteria.

## Materials and Methods

### Strains and UVC Disinfection Equipment

*Escherichia coli* (CMCC 44103), purchased from the Guangdong Microbiology Culture Center (Guangdong, China), was used as the test strain. *E. coli* was grown in Luria-Bertani (LB) broth at 37°C for 16 h till reached 10^9^ CFU mL^-1^ as determined using the nutritional agar (NA) count plating method at 37°C for 24 h. To simulate cell densities in drinking and reclaimed water system before UVC disinfection, 0.2 and 20 mL of bacterial culture were collected and washed with sterile saline solution (0.9%) three times by centrifugation (10000 rpm, 10 min) and re-suspended in 20 L of sterilized saline to obtain cell concentration of approximately 10^4^ CFU mL^-1^ (SDW) and 10^6^ CFU mL^-1^ (SRW), respectively.

**FIGURE 1:**
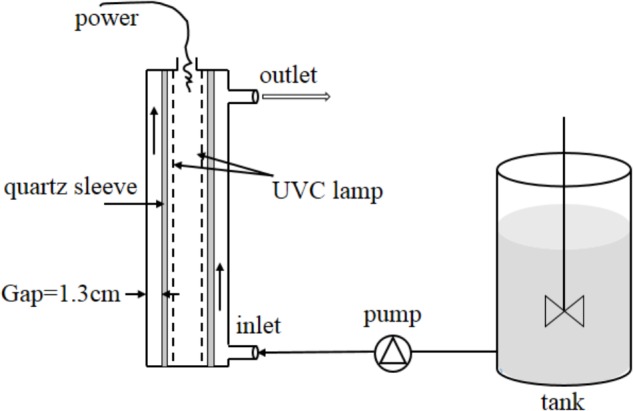
Schematic diagram of continuous-flow UVC disinfection unit. One low-pressure mercury arc lamp (254 nm, 14 W) mounted in a quartz sleeve running through the center of a chamber. The unit and lamp was covered by a stainless steel cylinder. The bacterial culture was pumped from the tank through a 1.3-cm annular gap between the inner surface of the chamber and the outer surface of the quartz sleeve.

A continuous-flow UVC disinfection unit (GMD-50A, Shuangyue, Beijing, China) was used in this study (**Figure 1**). This unit incorporated an annular stainless steel photoreactor (inner diameter = 4.5 cm and inner length = 24.5 cm), which contained a low-pressure mercury arc lamp (14 W, UVC efficiency = 36%, Philips, Netherlands) in the center of a quartz sleeve (outer diameter = 1.9 cm). The lamp was warmed up for 20 min before operation to achieve stable radiation. The bacterial culture was pumped from a reservoir through a 1.3-cm annular gap between the sleeve and the inner wall of the stainless steel reactor. The flow rates were set at 2.37, 5.55, 10.14, 21.20, and 34.12 cm s^-1^. By incorporating flow rate and geometry size of photoreactor to a Multiple Segment Source Summation (MSSS) model (Bolton, 2000), UVC doses were calculated to be 11.8, 18.9, 41.0, 73.8, and 172.2 mJ cm^-2^, respectively. The temperature of the cylinder after the addition of the bacteria and passage through the UVC apparatus remained constant at 20–22°C. All of the experiments were conducted in the dark.

### Culturable Cells Determined by HPC

To enhance the accuracy of experiment results, 1 mL rather than 100 μL of a UVC-treated bacterial solution was added to the plate medium, and the assay was performed on 10 plates in parallel with a control for each group. Three independent experiments were performed, and there were 10 replicates (plates)for each treatment in each independent experiment.

### Total Viable Cells Determined by CTC-FCM

Eight microliters of CTC (Thermo Fisher, United States) were added to 192 μL of simulated water with cell densities of 10^5^–10^6^ CFU mL^-1^ after UVC radiation. The solution was shaken for uniformity and incubated for 3 h at 37°C. CTC fluorescence intensity was determined by calculating the average florescence intensity per cell (average respiration strength)when the cells were subjected to FCM. FCM was performed using a Guava easyCyte 5 High Performance Flow Cytometer (Gernsheim, Germany) emitted with a fixed wavelength at 488 nm. Red fluorescence was detected at 635 nm. Voltage values for side angle light scatter (SSC) and red fluorescence were optimized using *E. coli* control cells. The total viable counts were measured by single staining with CTC. Three independent experiments were performed, and one sample from each treatment was prepared in each independent experiment.

### VBNC Cells Determined by HPC and CTC-FCM

Culturable cells were counted based on colonies from proliferation of single cell on plates. CTC-FCM can rapidly count variable cell at single-cell level. The number of VBNC *E. coli* was considered as the difference between the number of viable cells (CTC-FCM)and culturable cells (HPC). Ratio of cells in a VBNC state was considered as the number of VBNC cells divided by viable cells.

### Enrichment of Simulated Drinking Water and Simulated Reclaimed Water

Because the microbial biomass was low in SDW and SRW, bacteria first need to be enriched before DNA extraction and RNA isolation. Fifty milliliters of SDW (10 L for SRW) was filtrated through a standard vacuum manifold filtration system (JTFA0215, Jinteng, China), and the bacteria-containing filter membranes were used for the following experiments.

### Membrane Integrity Determined by PMA-qPCR

Five microliters of PMA (Biotium, Inc., Hayward, CA, United States) at a concentration of 1 mg mL^-1^ was added to 495 μL of sterile water to create a stock concentration of 20 μmol and stored at -20°C in the dark. The bacteria-containing filter membranes were submerged in each well of a 6-well plate with a working solution of PMA. After incubating for 5 min in the dark with occasional mixing, the samples were light-exposed for 15 min using a 650-W halogen light source (220V, 3400K, OSRAM, Munich, Germany). The 6-well plates were placed horizontally on ice and placed approximately 20 cm from the light source to avoid overheating. After photo-induced cross-linking, the bacteria-containing filter membranes were cut into pieces for DNA extraction. DNA was extracted from 1-mL samples using a Fast DNA Spin Kit (Tiangen, #DP302-02) following the manufacturer’s protocol. PCR targeting the 16S rRNA was performed using the forward primer 341F (5′-CCTACGGGAGGCAGCAG-3′) and reverse primer 534R (5′- ATTACCGCGGCTGCTGG-3′). Three independent experiments were performed, and one sample from each treatment was prepared in each independent experiment.

### Gene Expression Determined by RT-qPCR

An RNA isolation kit (PowerWater RNA Isolation Kit, Mo Bio, United States) was used to extract and purify total RNA from *E. coli* cells according to the manufacturer’s instructions. Total RNA was quantified spectrophotometrically based on optical density (OD) at 260/280 nm (AS-Nano100-1072; Aosheng, Shanghai, China). In the subsequent reverse transcription process, a FastQuant Reverse Transcription System (KR108, Tiangen Biotech, Beijing, China) was used for first-strand cDNA synthesis. To determine whether the cDNA product was amplified exclusively from RNA, a control without reverse transcriptase was included. Before proceeding with the qPCR, reverse transcriptase was inactivated using a heat block set at 95°C for 3 min. Three independent experiments were performed, and one sample from each treatment was prepared in each independent experiment.

### Quantification of DNA and cDNA by qPCR

qPCR was performed to amplify total DNA and cDNA using a Quant Studio 6 Flex Real-Time PCR System (Applied Biosystems) according to the manufacturer’s instructions: one cycle at 94°C for 30 s for predenaturation; 40 cycles at 94°C for 5 s and 60°C for 34 s; and then denaturation for one cycle. A reaction volume of 20 μL containing 10 μL2× Taq polymerase mix, 0.4 μL each forward and reverse primers (10 μmol), 7.2 μL double-distilled H_2_O, and 2 μL template was used for qPCR. The standard curve was constructed by a 10-fold serial dilution series of the plasmid, consequently, the results were correspondingly analyzed.

### Identification of Resuscitation by “Single-Cell Culture”

One hundred of 1.5 mL centrifuge tubes containing 10 μL of UVC-treated bacteria solution and 10 μL of LB medium were incubated at 37°C overnight. The number of tubes that became visibly turbid was counted and compared with the number of culturable cells after UVC treatment. Resuscitation was considered to occur if the former is more than the latter. This method was termed as “single cell culture.” Three independent experiments were performed, and one sample from each treatment was prepared in each independent experiment.

### Metabolic Activity Detected by D_2_O-Labeled Single-Cell Raman Spectroscopy

One milliliter of UVC-treated cell solution was introduced into sterilized tubes with 1 mL of LB broth and 1 mL of heavy water (D_2_O). The resulting D_2_O content was 33%, lower than the toxic dose of 50% at which growth inhibition occurred (Berry et al., 2015). The mixtures were then incubated at 37°C, 150 rpm and sampled at 0.5, 3, and 24 h for Raman spectroscopy, respectively. All of the cells were harvested by centrifuging at 5000 rpm for 3 min and then washed with sterile water twice to remove the culture medium. After washing, samples were spotted on aluminum-coated slides and air dried prior to Raman analysis. Single-cell Raman spectra ranging from 300 to 3300 cm^-1^ were obtained using a LabRAM ARAMIS (Horiba, Japan) confocal micro-Raman system equipped with a 300 g mm^-1^ grating. A 100× magnifying dry objective (NA = 0.90, Olympus) was used to observe and collect the Raman signal from a single cell. A total of around 60 Raman spectra from single cell with 20 from each of the three biological replicates of culture were acquired for each treatment. Raman results were from triplicate samples in one independent experiments. Raman spectra were preprocessed for baseline correction and normalization using LabSpec 5 software (Horiba, Japan). To quantify the degree of D substitution in C-H bonds (%C-D), the average intensities of C-D and C-H bands at 2040–2300 cm^-1^ and 2800–3100 cm^-1^ were used to calculate the CD/(CD + CH) intensity ratio. Box plot was used to depict distribution of CD/(CD + CH) intensity ratio from single cells.

### Statistical Analysis

A single-factor analysis of variance (ANOVA) was used to determine significant differences in the results of three independent (HPC, CTC-FCM, PMA-qPCR, and RT-PCR)or triplicate trials (Raman-D_2_O)using the SPSS 11.5 statistical software package. The results are expressed as means ± SE and were deemed statistically significant when *P*-values were less than 0.05.

## Results and Discussion

### Induction of VBNC State by a Continuous-Flow UVC Disinfection Equipment

Continue-flow UVC ranging from 0 to 172.2 mJ cm^-2^ of UV doses were used to simulate real-world UV disinfection in water treatment. Original *E. coli* concentration of about 10^4^ and 10^6^ CFU mL^-1^ were used to simulate the bacterial number in drinking and reclaimed water treatment, respectively. UVC-treated *E. coli* cells were then characterized by HPC and CTC-FCM to determine the cultivability, viability and VBNC state in SDW (**Table 1**) and SRW (**Table 2**).

**Table 1:** Culturability, viability, and VBNC state of *E. coli* after continue-flow UVC treatment in simulated drinking water (SDW).

UVC dose (mJ/cm^2^)	Culturable *E. coli* determined by HPC (Log CFU/mL)	*SD*	Viable *E. coli* CTC-FCM (Log cell/mL)	*SD*	VBNC *E. coli* (Log cell/mL)	Ratio of *E. coli* in a VBNC state
0	4.08	0.07	4.40	0.23	0.32	7.2%
11.8	2.40	0.28	4.78	0.45	2.38	49.8%
18.9	0.52	0.9	4.27	0.35	3.75	87.8%
41.0	none		4.61	0.48	4.61	100%
73.8	none		3.62	0.65	3.62	100%
172.2	none		3.17	0.13	3.17	100%

*SD is the standard deviation of results from three independent experiments. ‘None’ indicates there is no culturable cells*.

**Table 2:** Culturability, viability, and VBNC state of *E. coli* after continue-flow UVC treatment in simulated reclaimed water (SRW).

UVC dose (mJ/cm^2^)	Culturable *E. coli* determined by HPC (Log CFU/mL)	*SD*	Viable *E. coli* CTC-FCM (Log cell/mL)	*SD*	VBNC *E. coli* (Log cell/mL)	Ratio of *E. coli* in a VBNC state
0	6.22	0.27	6.64	0.47	0.22	3.3%
11.8	4.86	0.09	6.68	0.44	1.82	27.2%
18.9	2.50	0.06	6.65	0.51	4.15	62.4%
41.0	-0.33	0.37	6.63	0.47	6.63	∼100%
73.8	-1		6.45	0.34	6.45	∼100%
172.2	-1.15		6.35	0.26	6.35	∼100%

*SD is the standard deviation of results from three independent experiments*.

**Tables 1**, **2** clearly show that as the UVC dose increased, the number of culturable bacteria determined by HPC in both SDW and SRW decreased significantly. When UVC dose increased from 0 to 18.9 mJ cm^-2^, the number of cultural bacteria decreased from 4.08 to 0.52 log in SDW and from 6.22 to 2.5 log in SRW, respectively. The HPC results in SDW and SRW at UVC dose of 18.9 mJ cm^-2^ met the international standard for the total allowable number of bacterial cells in drinking water and reclaimed water (Sayre, 1988; Crook and Surampalli, 1996). When the UVC dose was higher than 18.9 mJ cm^-2^, there was no culturable bacteria in SDW; while in SRW, there was still small number of culturable bacteria corresponding to 0.47, 0.1, and 0.07 CFU mL^-1^ at UVC dose of 41, 73.8, and 172.2 mJ cm^-2^. These results indicated that UVC disinfection was highly efficient in reducing the culturability of bacteria.

For viable bacteria determined by CTC-FCM, no significant difference was observed in all UVC doses in both SDW (*P* = 0.13–0.65) and SRW (*P* = 0.26–0.51), indicating that all of cells were still capable of transporting electrons to retain respiratory capacity. Active respiration, as an important feature of viable bacteria, enables bacteria to obtain energy by oxidizing and decomposing the organic matter. Previous studies also indicated that UVC radiation did not affect the respiration of bacteria except at extraordinarily high dose.

For instances, Blatchley et al. (2001) found that 100 mJ cm^-2^ of UVC radiation did not decrease the number of respiring *E. coli* by directly measuring oxygen intake. Lazarova et al. (1998) revealed that UVC radiation at 46 mJ cm^-2^ decreased the CFU of fecal coliforms by 3.5 log, whereas only small changes in CTC count were observed by epifluorescence microscopy. Lau et al. (2003) showed that only a very high UVC radiation of 750 and 1500 mJ cm^-2^ can significantly reduce the percentage of respiring *Roseobacter litoralis*; while 15 mJ cm^-2^ of UVC radiation did not affect cell respiration. These results were consistent with our findings and it can be concluded that UVC disinfection did not decrease the respiratory activity of certain bacteria at the doses used in water treatment plants (usually less than 100 mJ cm^-2^). This is different from chlorine treatment, which decreases *E. coli* respiration at the doses used in the drinking water treatment process (Lau et al., 2003). Combined with the fact that the culturability of *E. coli* decreased (in SRW) or even lost (in SDW) with UVC dose increasing, continuous-flow UVC treatment can thus be fully demonstrated to induce *E. coli* into a VBNC state in both SDW and SRW.

The number of bacteria entering into VBNC state was calculated by subtracting the number of HPC-determined culturable cell from that of CTC-FCM-determined viable cell. The value difference was then divided by CTC-FCM number to obtain the ratio of VBNC cells. **Tables 1**, **2** show that as the UVC dose increases, bacteria that enter a VBNC state account for more and more of the total viable bacteria until reaching 100%, indicating that UVC irradiation induced more cells into VBNC state. For control group without UVC irradiation, viable cells are more than culturable cell in both SDW and SRW. VBNC bacteria in the control were determined to be 0.32 and 0.22 log, accounting for 7.2 and 3.3% of the total viable bacteria in SDW and SRW, respectively. The larger ratio than zero might illustrate that though without any environment press stimulation, parts of *E. coli* still could not grow on the NA plate for their lower activity or a slow growth rate, and which could be classified as VBNC bacteria.

### Membrane Integrity and Gene Expression of VBNC Bacteria

**FIGURE 2:**
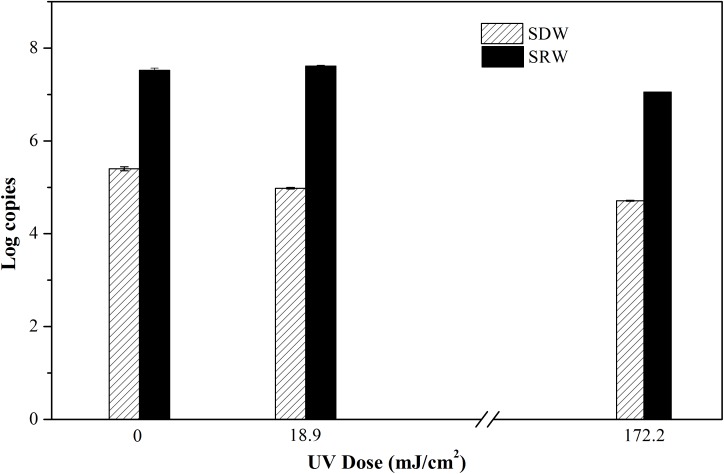
Log copies of 16S rRNA gene quantifying viable cells with integral membrane of *E. coli* after treatment at UVC doses of 0, 18.9, and 172.2 mJ/cm^2^ were determined by propidium monoazide quantitative polymerase chain reaction (PMA-qPCR). Mean values with SD from three independent measurements were shown. SDW, simulated drinking water; SRW, simulated reclaimed water.

In order to further understand bacteria in a VBNC state, molecular biological methods including PMA-qPCR and RT-qPCR were used to characterize membrane integrity and gene expression, respectively. PMA is a dye that can penetrate membrane-damaged cells and binds to their DNA. PMA-bound DNA cannot be amplified in subsequent PCR. PMA combined with PCR is often used to determine cellular integrity (Zhang and Yu, 2010). In this study, PMA was cross-linked to DNA in cells following continuous-flow UVC radiation. 16S rRNA was then amplified and quantified, the resulting gene copy number was used to indicate cells with intact membrane. PMA-qPCR reveals that there were no decrease in 16S rRNA copy number even at a high UVC dose of 172.2 mJ/cm^2^ (**Figure 2**), indicating that the membrane integrity of VBNC bacteria was maintained (*P* = 0.054 and 0.051 for SDW and *P* = 0.073 and 0.38 for SRW). UVC light has been demonstrated before to transform oxygen molecules in bacterial liquids into ozone (Bale et al., 2008), which can further produce hydroxyl radicals (OH⋅) (Atkinson et al., 1992). Both ozone and OH⋅; can degrade organic compounds such as bacterial cell walls, cell membranes, and protein (Lunov et al., 2014). Despite the damage potential of UVC, rupture of cell membrane even at UVC dose as high as 172.2 mJ cm^-2^ was not observed, possibly due to the insufficient amount of ozone or hydroxyl radical generated in this study.

**FIGURE 3:**
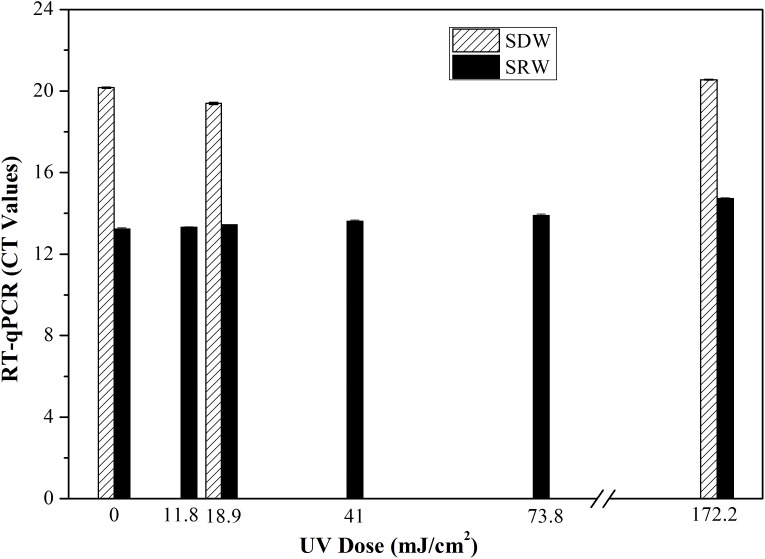
Transcription or gene expression level of 16S rRNA of *E. coli* represented as Ct values after treatment with different UVC doses based on RT-qPCR. Mean values with standard deviation from three independent measurements were shown. SDW, simulated drinking water; SRW, simulated reclaimed water.

Only live bacteria can transcribe RNA, so the production of RNA is another indicator of bacterial viability in a VBNC state (Lleò et al., 2000). Here the level of 16S rRNA transcripts from *E. coli* after continuous-flow UVC treatment was determined using RT-qPCR (**Figure 3**). No significant effect on Ct values representing transcription level of 16S rRNA (SDW, *P* = 0.324–0.348; SRW, *P* = 0.185–0.933) was observed under UVC doses ranging from 0 to 172.2 mJ cm^-2^, indicating that UVC could not damage the expression level of 16S rRNA.

Overall, there was no significant difference between UVC-treated samples and control based on the results of PMA-qPCR, RT-qPCR, and CTC-FCM, indicating membrane integrity, gene expression level and respiratory activity of VBNC bacteria were not significantly affected by UVC treatment. This is different from other disinfectants such as oxidizing chlorine or ultrasound, wherein significant differences in treated and untreated sample were found using PMA-qPCR and RT-qPCR assay (Zhang and Yu, 2010; Lin et al., 2017). The reason should be related to the different disinfection mechanisms. UVC disinfection generally maintains cellular integrity because it targets microbial DNA inside the cells, while chlorine or ultrasound oxidize the bacterial cell and break the cellular membranes.

### Identification of Resuscitated VBNC Bacteria

If bacteria were maintained in a VBNC or dormant state forever, there might be no threat to human health, for many pathogenic mechanisms do not function in a VBNC state (Oliver and Bockian, 1996; Sun et al., 2008). However, VBNC bacteria can find ways to resuscitate under specific stimuli such as nutrient. Monitoring resuscitation is important for assessing the threat of VBNC bacteria to the environment. Based on the definition of resuscitation, only bacteria that have entered a VBNC state and re-grown can be called resuscitated (Xu et al., 1982). Residual culturable cells showing normal growth after UVC treatment should not be classified as resuscitated.

**FIGURE 4:**
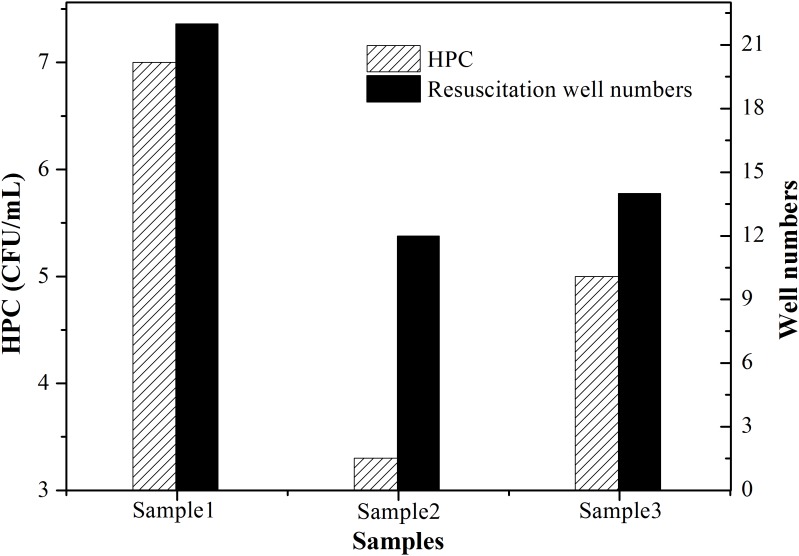
Resuscitation identified in simulated reclaimed water (SRW) after treatment by 41 mJ cm^-2^ UVC. One hundred of 1.5 mL centrifuge tubes containing UVC-treated bacteria solution and LB medium were incubated at 37°C overnight in triplicate. Patterned column denoted as ‘HPC’ represents number of culturable cells determined by HPC; black column denoted as ‘Resuscitation tube numbers’ represents the number of tubes that became visibly turbid.

In this study, single cell culture was used to identify resuscitated bacteria. Its principle and result are described as follows by taking SRW as an example. After SRW was treated by UVC dose of 41.0 mJ cm^-2^ in triplicate, the number of culturable bacteria was 7, 3.3, and 5 CFU mL^-1^, respectively. 1 mL of this bacterial solution was then divided into 100 portions, and only a small portion of no more than 7, 3.3, and 5% can be seeded with one culturable cell. After incubation in rich medium such as LB, if the number of tubes with turbid bacterial suspension was more than that of the culturable bacteria after UVC treatment, VBNC cells were considered to have resuscitated. The number of HPC-determined culturable cells (HPC) and tube number with turbid bacteria (including both culturable and resuscitated cells) were shown in **Figure 4**. All triplicate tests indicated that the number of tubes becoming visible turbid was more than that of culturable bacteria, demonstrating that the UVC-induced VBNC bacteria resuscitated during post-incubation. Considering that CFU per milliliter is used in International System of Units to count bacterial colonies, dividing each milliliter of UVC-treated water sample into 100 portions is a reasonable way to identify resuscitation. However, single-cell culture method is time- and labor-consuming, it is not suitable when a lot of samples are required to assay. In addition, although the nutritional level of resuscitation solution is higher than reclaimed and drinking water, it is similar to the nutritional level of human intestinal environment, based on comparison of total organic carbon (TOC).

### Changes of Metabolic Activity in VBNC Bacteria During Post-incubation by D_2_O-Labeled Raman Spectroscopy at the Single-Cell Level

Evolution of metabolic activity of UVC-treated bacteria during 24 h post-incubation was studied by D_2_O-labeled Raman spectroscopy at the single-cell level. The principle of detection is based on the fact that metabolic activity is proportional to the level of D_2_O-derived D from D_2_O incorporated into active cells, and the resulting C-D bonds can be sensitively detected by single-cell Raman spectroscopy. The intensity ratio of (C-D)/(C-H + C-D) allows semi-quantitative measurement of bacterial metabolic activity without prior knowledge of physiological status (Berry et al., 2015; Lorenz et al., 2017; Song et al., 2017; Tao et al., 2017). Single-cell level investigation also allows revealing heterogeneous metabolic activity of bacteria at a high resolution.

Based on the key time points for discharging UVC-disinfected water, UVC-treated cells were incubated with D_2_O-amended medium for 0.5 h (the hydraulic detention time in a clear water tank), 3 h (the time when water flows from the outlet to the point of use nearby), and 24 h (the time when water flows from the outlet to distant points of use). The obtained information on changes of metabolic activity will allow monitoring the recovery of UVC-treated cells, an important aspect related to disinfection efficacy and thus health risk.

**FIGURE 5:**
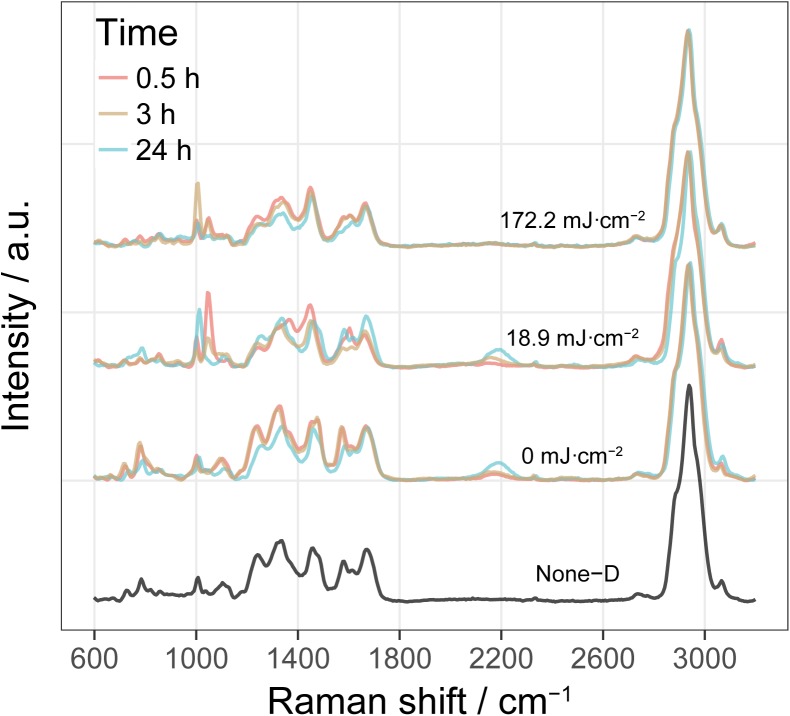
Single-cell Raman spectra of *E. coli* in simulated reclaimed water (SRW) after incubation with D_2_O-amended medium for 0.5, 3, and 24 h pre-treated by UVC dose of 0, 18.9, and 172.2 mJ cm^-2^. Spectrum labeled as None-D is from *E. coli* grown in D_2_O-free medium. Each Raman spectrum shown here is an average of around 60 spectra from single cell with 20 from each of the three biological replicates of culture.

**Figure 5** shows typical single-cell Raman spectra of *E. coli* after incubation with D_2_O-amended medium for 0.5, 3, and 24 h pre-treated by UVC dose of 0, 18.9, and 172.2 mJ cm^-2^. No visible C-D peak was observed in D_2_O-free cultures (None-D). By comparison, cells grown in D_2_O-amended medium show a clear C-D band centered at around 2200 cm^-1^. The intensity of C-D band increased with incubation time for cells without UVC treatment (0 mJ cm^-2^) or with a low UVC dose of 18.2 mJ cm^-2^, indicating increasing incorporation of D and substitution of C-H bonds with C-D bonds in these metabolic active cells. By comparison, cells exposed to a high UVC dose of 172.2 mJ cm^-2^ did not show a clear C-D band even after 24 h incubation, indicating that UVC greatly impaired cells and induced an irrecoverable loss of metabolic activity.

**FIGURE 6:**
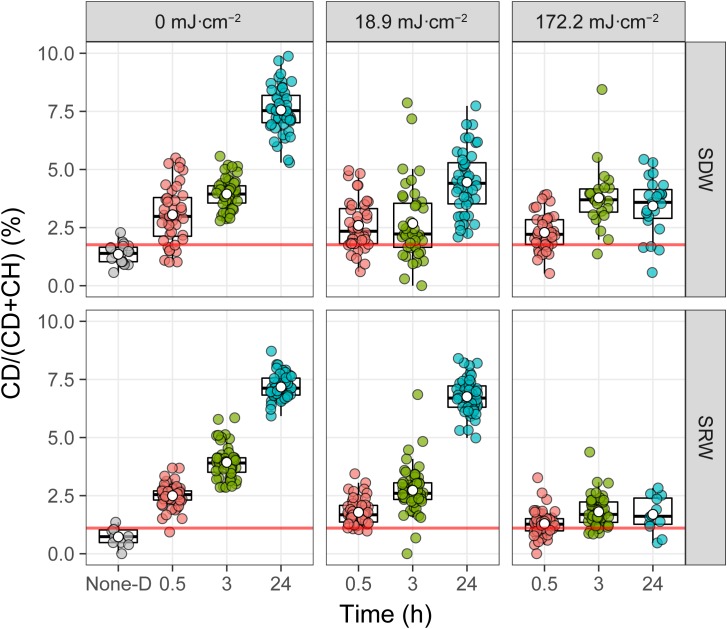
Intensity of deuterium incorporation in single *E. coli* measured by %CD. Single-cell spectra are denoted by points, and population quartiles are shown as box plots. The red line is the threshold for considering a cell labeled by D. It is determined by the upper quantile of boxplot from the bacterial sample without incubation with D_2_O (None-D indicated in the x axis).

**Figure 6** shows the UVC dose- and time-dependent C-D band ratio of randomly selected individual cells in SDW and SRW. At a UVC dose of 0 mJ cm^-2^, C-D ratio after 0.5 h incubation was significantly higher than that without D_2_O (None-D) (*P* < 0.001) in both SDW and SRW, and continued to increase from 0.5 to 24 h, indicating that normal metabolic active cells can rapidly and increasingly incorporate D_2_O with time. When UVC dose was increased to 18.9 mJ cm^-2^, after 0.5 h of incubation, C-D ratio of cells in both SDW (*P* < 0.001) and SRW (*P* < 0.001) was lower than that without UVC treatment, indicating that UVC treatment impaired bacteria and decreased their metabolic activity. After 3 h of incubation, C-D ratio of cells in SDW was close to that at 0.5 h, but increased significantly in SRW (*P* < 0.001), indicating a faster recovery of metabolic activity of cells in SRW than that in SDW. After incubation for 24 h, C-D ratio profile in SRW was almost identical to that of the control, indicating that UVC-impaired cells in SRW has recovered their metabolic activity to a level similar to control. By comparison, C-D ratio in SDW also increased, but to a level significantly lower than that of control or SRW (*P* < 0.001), revealing a slower recovery of metabolic active cells in SDW than in SRW. Considering that there are still a certain number of culturabe cells in SDW and SRW after exposed to 18.9 mJ cm^-2^, metabolic active cells during post-incubation detected by D_2_O-labeled Raman could origin from both culturable cells and VBNC state cells. The culturable cells in SRW was two orders of magnitude more than that in SDW, accounting more for the highly metabolic active cells during post-incubation (**Tables 1**, **2**).

When the UVC dose was increased to 172.2 mJ cm^-2^, despite the fact that the number of culturable bacteria in both SDW and SRW were almost zero (**Tables 1**, **2**), Raman spectroscopy results indicated that some bacteria still held a certain level of metabolic activity, as observed by the significantly higher C-D ratio in cells than that without D_2_O amendment (*P* < 0.001). However, compared with control or that treated with a lower 18.9 mJ cm^-2^ UVC dose, the metabolic activity of cells treated by 172.2 mJ cm^-2^ UVC in both SDW (*P* < 0.002) and SRW (*P* < 0.001) was lower. In addition, with incubation time increasing, the metabolic activity just recovered slightly even after 24 h. These facts indicated that a higher UVC dose induced a more serious impairment on bacteria and thus a decreased ability to recover metabolic activity during post-incubation. Because almost no culturable cells were detected at UVC dose of 172.2 mJ cm^-2^, VBNC state cells instead of culturable cells were deemed to recover metabolic activity during post-incubation and account for the small increase of C-D ratio.

Single-cell D_2_O-labeled Raman spectroscopy used here revealed a UVC dose- and incubation time-dependent metabolic activity of cells in a semi-quantitative way. A lower UVC dose, longer post-incubation time, and higher initial number of bacteria were demonstrated to result in a larger degree of recovery of metabolic activity. In addition, metabolic heterogeneity at the single-cell level was also revealed by single-cell D_2_O-labeled Raman spectroscopy. Heterogeneous responses is an important strategy for populations to adapt to fluctuating environments, even for genetically identical population (Şimşek and Kim, 2018). Identification of the subpopulation with higher metabolic activity is very important in evaluating the efficiency of UVC disinfection and the potential risk of resuscitation.

This work demonstrates the sensitivity and rapidness of D_2_O-labeled Raman capable of detecting D incorporation after only 30 min incubation. Another advantage of D_2_O-Raman is that it is a truly phenotypic characterization of metabolic active cells, and metabolic heterogeneity can be revealed at a high-resolution single-cell level. The limitation is the low throughput compared with fluorescence-based method such as flow cytometry due to the relative weak Raman signal of cells. In addition, for UV-treated cell containing both VBNC and culturable cells, D_2_O-labeled Raman can not distinguish them.

## Conclusion

To address the problem whether disinfection technologies extensively used in water treatment could induce bacteria into a silent but potentially dangerous VBNC state and related recovery processes, a continuous-flow UVC simulating real-world UVC disinfection in both drinking and reclaimed water treatment plants was designed. By using this setup, UVC disinfection was demonstrated to induce *E. coli* into a VBNC state by HPC and CTC-FCM assay. The ratios of VBNC cells were indicated to increase with UVC dose. Membrane integrity and transcript level of VBNC bacteria were demonstrated to be unaffected by UVC treatment. Resuscitation of VBNC cells was also identified using a new single cell culture method. D_2_O-labeled Raman spectroscopy was first introduced to study metabolic activity evolution of UVC-treated bacteria during post-incubation. A lower UVC dose, longer post-incubation time, and higher initial number of bacteria were demonstrated to result in a larger degree of recovery of metabolic activity. Subpopulation with higher metabolic activity potentially was also revealed by single-cell Raman. This work provides a comprehensive evaluation of VBNC cells associated with water disinfection and clearly indicates the potential risk of VBNC cells from UVC disinfection process. In the future, it is important to focus on more features of VBNC bacteria, particularly pathogenicity and infectivity that is closely related to human health.

## Author Contributions

SZ, XY, and LC conceived and designed the project. CY and SC performed the molecular biology experiments. LG and KY performed the Raman experiments. SZ, LG, KY, YZ, and LC analyzed the data. SZ and LC wrote the manuscript. YZ and WH revised the paper. All authors read and approved the final manuscript.

## Conflict of Interest Statement

The authors declare that the research was conducted in the absence of any commercial or financial relationships that could be construed as a potential conflict of interest.

## References

[B1] Atkinson R. Aschmann S. M. Arey J. Shorees B. (1992). Formation of OH radicals in the gas phase reactions of O3 with a series of terpenes. *J. Geophys. Res.* 97 6065–6073. 10.1029/92JD00062

[B2] Bale C. S. E. Ingham T. Commane R. Heard D. E. Bloss W. J. (2008). Novel measurements of atmospheric iodine species by resonance fluorescence. *J. Atmos. Chem.* 60 51–70. 10.1007/s10874-008-9108-z

[B3] Belosevic M. Craik S. A. Stafford J. L. Neumann N. F. Kruithof J. Smith D. W. (2001). Studies on the resistance/reactivation of *Giardia muris* cysts and cryptosporidium parvum oocysts exposed to medium-pressure ultraviolet radiation. *FEMS Microbiol. Lett.* 204 197–203. 10.1111/j.1574-6968.2001.tb10885.x 11682201

[B4] Berry D. Mader E. Lee T. K. Woebken D. Wang Y. Zhu D. (2015). Tracking heavy water (D2O) incorporation for identifying and sorting active microbial cells. *Proc. Natl. Acad. Sci. U.S.A.* 112 E194–E203. 10.1073/pnas.1420406112 25550518 PMC4299247

[B5] Blatchley E. R. Dumoutier N. Halaby T. N. Levi Y. Laine J. M. (2001). Bacterial responses to ultraviolet irradiation. *Water Sci. Technol.* 43 179–186. PMID: 11436779 10.2166/wst.2001.0614 11436779

[B6] Bolton J. R. (2000). Calculation of ultraviolet fluence rate distributions in an annular reactor: significance of refraction and reflection. *Water Res.* 34 3315–3324. 10.1016/S0043-1354(00)00087-7

[B7] Crook J. Surampalli R. Y. (1996). Water reclamation and reuse criteria in the US. *Water Sci.Technol.* 33 451–462. 10.1111/j.1574-6968.1994.tb06662.x

[B8] Cui L. Yang K. Li H. Z. Zhang H. Su J. Q. Paraskevaidi M. (2018). Functional single-cell approach to probing nitrogen-fixing bacteria in soil communities by resonance raman spectroscopy with (15)N2 labeling. *Anal. Chem.* 90 5082–5089. 10.1021/acs.analchem.7b05080 29557648

[B9] Cui L. Yang K. Zhou G. Huang W. E. Zhu Y. G. (2017). Surface-enhanced Raman spectroscopy combined with stable isotope probing to monitor nitrogen assimilation at both bulk and single-cell level. *Anal. Chem.* 89 5793–5800. 10.1021/acs.analchem.6b04913 28452221

[B10] Huang W. E. Griffiths R. I. Thompson I. P. Bailey M. J. Whiteley A. S. (2004). Raman microscopic analysis of single microbial cells. *Anal. Chem.* 76 4452–4458. 10.1021/ac049753k 15283587

[B11] Jiang Q. Fu B. Chen Y. Wang Y. Liu H. (2013). Quantification of viable but nonculturable bacterial pathogens in anaerobic digested sludge. *Appl. Microbiol. Biot.* 97 6043–6050. 10.1007/s00253-012-4408-2 22996281

[B12] Junge K. Eicken H. Deming J. W. (2004). Bacterial activity at -2 to -20°C in Arctic wintertime sea ice. *Appl. Environ. Microbiol.* 70 550–557. 10.1128/AEM.70.1.550-557.2004 14711687 PMC321258

[B13] Kaprelyants A. S. Mukamolova G. V. Kell D. B. (1994). Estimation of dormant Micrococcus luteus cells by penicillin lysis and by resuscitation in cell-free spent culture medium at high dilution. *FEMS Microbiol. Lett.* 115 347–352. 10.1111/j.1574-6968.1994.tb06662.x

[B14] Lau S. C. Harder T. Qian P. Y. (2003). Induction of larval settlement in the serpulidpolychaeteHydroideselegans (Haswell): role of bacterial extracellular polymers. *Biofueling* 19 197–204. 10.1080/08927014.2003.10382982 14619288

[B15] Lazarova V. Savoye P. Janex M. L. Blatchley E. R. Pommepuy M. (1998). Advanced wastewater disinfection technologies: state of the art and perspectives. *Water Sci. Technol.* 40 203–213. 10.2166/wst.1999.0593

[B16] Li D. Zeng S. Gu A. Z. He M. Shi H. (2013). Inactivation, reactivation and regrowth of indigenous bacteria in reclaimed water after chlorine disinfection of a municipal wastewater treatment plant. *J. Environ. Sci.* 25 1319–1325. 10.1016/S1001-0742(12)60176-4 24218842

[B17] Li M. Q. Canniffe D. P. Jackson P. J. Davison P. A. FitzGerald S. Dickman M. J. (2012). Rapid resonance Raman microspectroscopy to probe carbon dioxide fixation by single cells in microbial communities. *ISME J.* 6 875–885. 10.1038/ismej.2011.150 22113377 PMC3309358

[B18] Lin H. Ye C. Chen S. Zhang S. Yu X. (2017). Viable but non-culturable *E.coli* induced by low level chlorination have higher persistence to antibiotics than their culturable counterparts. *Environ. Pollut.* 230 242–249. 10.1016/j.envpol.2017.06.047 28662489

[B19] Lleò M. M. Benedetti D. Tafi M. C. Signoretto C. Canepari P. (2007). Inhibition of the resuscitation from the viable but non-culturable state in *Enterococcus faecalis*. *Environ. Microbiol.* 9 2313–2320. 10.1111/j.1462-2920.2007.01345.x 17686027

[B20] Lleò M. M. Pierobon S. Tafi M. C. Signoretto C. Canepari P. (2000). mRNA detection by reverse transcription-PCR for monitoring viability over time in an *Enterococcus faecalis* viable but nonculturable population maintained in a laboratory microcosm. *Appl. Environ. Microbiol.* 66 4564–4567. 10.1128/AEM.66.10.4564-4567.2000 11010918 PMC92344

[B21] Lorenz B. Wichmann C. Stockel S. Rosch P. Popp J. (2017). Cultivation-free Raman spectroscopic investigations of bacteria. *Trends Microbiol.* 25 413–424. 10.1016/j.tim.2017.01.002 28188076

[B22] Lunov O. Zablotskii V. Churpita O. Chánová E. Syková E. Dejneka A. (2014). Cell death induced by ozone and various non-thermal plasmas: therapeutic perspectives and limitations. *Sci. Rep.* 4:7129. 10.1038/srep07129 25410636 PMC4238021

[B23] Manina G. McKinney J. D. (2014). A Single-Cell Perspective on Non-Growing but Metabolically Active (NGMA) Bacteria. *Pathog. Mycobacterium Tuberc. Its Interact. Host Organ.* 374 135–161. 10.1007/82-2013-333 23793585

[B24] Moreno Y. Piqueres P. Alonso J. L. Jiménez A. González A. Ferrús M. A. (2007). Survival and viability of *Helicobacter* pylori after inoculation into chlorinated drinking water. *Water Res.* 41 3490–3496. 10.1016/j.watres.2007.05.020 17585990

[B25] Oliver J. D. (2000). “The public health significance of viable but nonculturable bacteria,” in *Nonculturable Microorganisms in the Environment*, eds Colwell R. R. Grimes D. J. (Washington, DC: ASM Press), 277–300.

[B26] Oliver J. D. Bockian R. (1996). In vivo resuscitation, and virulence towards mice, of viable but nonculturable cells of Vibrio vulnificus. *Appl. Environ. Microb.* 61 2620–2623. 7618873 10.1128/aem.61.7.2620-2623.1995 PMC167533

[B27] Oliver J. D. Dagher M. Linden K. (2005). Induction of *Escherichia coli* and *Salmonella typhimurium* into the viable but nonculturable state following chlorination of wastewater. *J. Water Health* 3 249–257. 10.2166/wh.2005.040 16209029

[B28] Orta de Velásquez M. T. YáñezNoguez I. Casasola Rodríguez B. Román Román P. I. (2017). Effects of ozone and chlorine disinfection on VBNC *Helicobacter pylori* by molecular techniques and FESEM images. *Environ. Technol.* 38 744–753. 10.1080/09593330.2016.1210680 27432258

[B29] Sayre I. M. (1988). International standards for drinking water. *J. Am. Water Works Assoc.* 80 53–60. 10.1002/j.1551-8833.1988.tb02980.x

[B30] Şimşek E. Kim M. (2018). The emergence of metabolic heterogeneity and diverse growth responses in isogenic bacterial cells. *ISME J.* 12 1199–1209. 10.1038/s41396-017-0036-2 29335635 PMC5932066

[B31] Slimani S. Robyns A. Jarraud S. Molmeret M. Dusserre E. Mazure C. (2012). Evaluation of propidium monoazide (PMA) treatment directly on membrane filter for the enumeration of viable but non cultivable Legionella by qPCR. *J. Microbiol. Methods* 88 319–321. 10.1016/j.mimet.2011.12.010 22212760

[B32] Song Y. Cui L. Lopez J. A. S. Xu J. Zhu Y. G. Thompson I. P. (2017). Raman-deuterium isotope probing for in-situ identification of antimicrobial resistant bacteria in Thames River. *Sci. Rep.* 7:16648. 10.1038/s41598-017-16898-X 29192181 PMC5709456

[B33] Sun F. Chen J. Zhong L. Zhang X. H. Wang R. Guo Q. (2008). Characterization and virulence retention of viable but nonculturableVibrioharveyi. *FEMS Microbiol. Ecol.* 64 37–44. 10.1111/j.1574-0941.2008.00442.X 18248440

[B34] Tao Y. F. Wang Y. Huang S. Zhu P. F. Huang W. E. Ling J. Q. (2017). Metabolic-activity-based assessment of antimicrobial effects by D2O-labeled single-cell Raman microspectroscopy. *Anal. Chem.* 89 4108–4115. 10.1021/acs.analchem.6b05051 28282113

[B35] Wang Y. Huang W. E. Cui L. Wagner M. (2016). Single cell stable isotope probing in microbiology using Raman microspectroscopy. *Curr. Opin. Biotechnol.* 41 34–42. 10.1016/j.copbio.2016.04.018 27149160

[B36] Whitesides M. D. Oliver J. D. (1997). Resuscitation of *Vibrio vulnificus* from the viable but nonculturable state. *Appl. Environ. Microbiol.* 63 1002–1005. 10.1128/jb.173.16.5054-5059.1991 16535534 PMC1389128

[B37] Xu H. S. Roberts N. Singleton F. Attwell R. Grimes D. Colwell R. (1982). Survival and viability of nonculturable *Escherichia coli* and *Vibrio cholerae* in the estuarine and marine environment. *Microb. Ecol.* 8 313–323. 10.1007/BF02010671 24226049

[B38] Zhang S. Yu X. (2010). PMA in combination with quantitative PCR for the detection of inactivation efficacy by ultrasonic sonication. *Fresenius Environ. Bull.* 19 940–944.

[B39] Zhang S. H. Ye C. S. Lin H. R. Lv L. Yu X. (2015). UVC disinfection induces a VBNC state in *Escherichia coli* and *Pseudomonas aeruginosa*. *Environ. Sci. Technol.* 49 1721–1728. 10.1021/es505211e 25584685

